# The Pathogenesis of Chiari Malformation and Syringomyelia: A Case Report and Systematic Review of Current Theories

**DOI:** 10.7759/cureus.47301

**Published:** 2023-10-19

**Authors:** Priya Sivakumaran, Neil Ashwood, Muhammad Kamal, Nithish Jayakumar

**Affiliations:** 1 Geriatrics, Kingston Hospital National Health Service (NHS) Foundation Trust, London, GBR; 2 Trauma and Orthopaedics, University Hospitals of Derby and Burton National Health Service Foundation Trust, Derby, GBR; 3 Neurology, Royal Victoria Infirmary, Newcastle upon Tyne Hospitals, Newcastle upon Tyne, GBR; 4 Neurosurgery, The James Cook University Hospital, Middlesbrough, GBR

**Keywords:** evidence-based anatomy, surgical and clinical management, clinical case report, syringomyelia, chiari malformations

## Abstract

We report a case of a 42-year-old female presenting with left axillary pain radiating down the arm and weakness in the ipsilateral hand. Specialist examinations of neurological and musculoskeletal systems were insignificant. Magnetic resonance imaging (MRI) of the whole spine and brain revealed cerebellar tonsillar herniation of 9-10mm indicating a Chiari type 1 malformation and a large tubular T2 high-intensity lesion in the cervical cord, extending from the C2/3-disc level down to C6/C7 as well as a similar but smaller lesion behind the bodies of C7 and T1. Both lesions were consistent with syringomyelia. Surgical intervention was deemed inappropriate, and she was treated with three months of physiotherapy. Regular follow-up for two years showed gradual symptom resolution, syrinx shrinkage, and no further complications arising secondary to Chiari type 1 malformation.

Chiari malformation is an anatomical anomaly of the cranio-cervical junction. It is often incidentally found on MRI, but although asymptomatic in the population, complications associated with the condition such as syringomyelia are a common initial presentation. The relationship between Chiari malformation, particularly Chiari type 1 malformation, and syringomyelia is close with the majority of patients often presenting with idiopathic syringomyelia also found to have a Chiari type 1 malformation. Considerable discussion about the pathogenic mechanisms for syringomyelia development in Chiari malformation is recognized and advancing continually.

## Introduction

In 1891, Han Chiari initially described three types of cerebellar deformity defining them as “alterations in the cerebellum resulting from cerebral hydrocephalus” [1]. He then in a subsequent paper added a fourth type, cerebellar hypoplasia [2]. These cerebellar deformities, now known as “Chiari malformations”, are today described as an abnormality of hindbrain descent resulting in a structural variation of the cerebellum and the skull base. Hydrocephalus is no longer considered a defining characteristic of these hindbrain malformations.

The most common type of Chiari malformation is Chiari type 1. Chiari type 1 involves the displacement of the cerebellar tonsils up to 5mm caudally below the foramen magnum into the upper cervical canal with no associated defects of the brainstem or supratentorial regions [3]. Chiari type 2 malformation is rarer than Chiari type 1 but more prevalent than Chiari type 3 (rarest) and 4. It involves more significant cerebellar herniation with a displacement of the inferior vermis and the brainstem into the upper cervical canal [4]. The severity of hindbrain descent and structural abnormality of the cerebellum and skull base is more significant in Chiari types 3 and 4, and these types are associated with a high morbidity and mortality of all Chiari subtypes.

Clinical presentations of Chiari malformations can vary significantly depending on the severity of hindbrain herniation. Syringomyelia is an important presenting feature of Chiari malformation and commonly affects the lower cervical or upper thoracic spinal cord [5]. This can present in a variety of different ways but commonly a bilateral cape-like sensation of loss of pain and temperature sense is seen with associated muscle weakness and atrophy in the hands and the arms. This occurs as the expanding CSF-filled syrinx compresses the spinothalamic tract neurons decussating in the anterior white commissure, whilst the posterior columns are spared. Sensory symptoms include paraesthesia and hyperaesthesia, with muscular weakness, particularly of the intrinsic muscle of the hand. Spasticity in the lower limbs can also occur where there is syrinx expansion and compression of the adjacent lateral corticospinal tracts. 

## Case presentation

**Figure 1:**
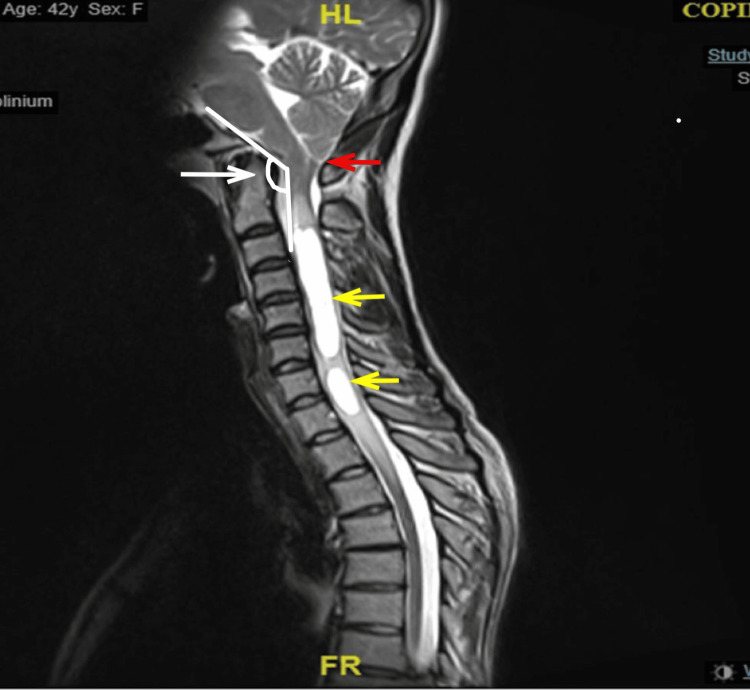
MRI of the patient showing cerebellar tonsillar herniation and distortion of the medulla highlighted by the red arrow. Two high-intensity lesions, highlighted by yellow arrows, are seen largely within the cervical cord. The clival canal angle is denoted by the white arrow.

A 42-year-old female presented with left axillary pain radiating down the arm and weakness in the ipsilateral hand. She did not report any motor weakness of the lower limbs, headache, facial weakness, or gait and balance problems. There was no further noted history of trauma, preceding viral illness, systemic involvement, or previous neurological/muscular-skeletal conditions. A detailed specialist examination revealed no focal neurological deficit and a functional musculoskeletal system. MRI of the whole spine and brain revealed cerebellar tonsillar herniation of 9-10mm highlighting Chiari type 1 malformation, with no associated hydrocephalus or syringobulbia. The clival canal angle was measured at 135°. The patient was also noted to have a large tubular T2 high-intensity lesion in the cervical cord, extending from the C2/3-disc level down to C6/C7 as well as a similar but smaller lesion behind the bodies of C7 and T1 (Figure 1). Both lesions were consistent with syringomyelia. There was no associated neurological impingement of the cervical nerve roots, (although irritation of the cervical nerve roots could not be excluded) or signs of significant degeneration of the cervical spine and lateral recess narrowing. Normal curvature and alignment of the spine were present with no evidence of spina bifida or mass lesions in the spinal canal. Her symptoms were consistent with a diagnosis of cervical radiculopathy secondary to syringomyelia and Chiari type 1 malformation. The lack of significant neurological compromise and substantial structural abnormality meant surgical intervention was not necessary and she was treated with education and a physiotherapy program including manual therapy and therapeutic exercise techniques. Shunt placement was also not considered as the overall risk of the procedure was greater than the added benefit due to the minimal neurological compromise seen. Following three months of physiotherapy, she noticed her symptoms resolved. She was followed up regularly for two years to monitor syrinx progression, the development of any other neurological symptoms, and complications of Chiari malformation. She was symptom-free for the complete follow-up, with evidence of significant syrinx shrinkage and no other complications of Chiari malformation developing.

Systemic literature review 

**Figure 2:**
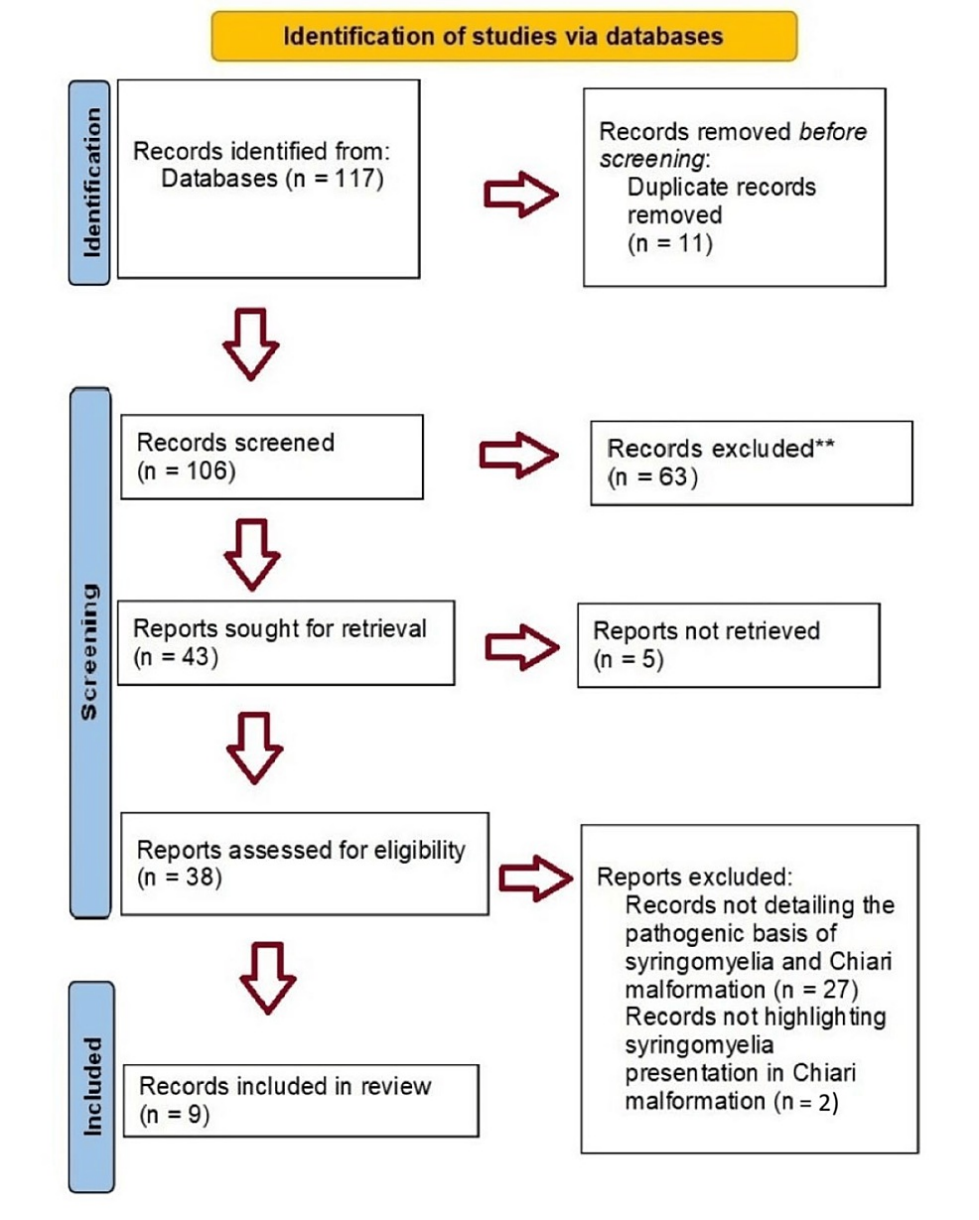
PRISMA flowchart highlighting selection of articles. PRISMA: Preferred Reporting Items for Systematic Reviews and Meta-Analyses

A literature search was performed following Preferred Reporting Items for Systematic Reviews and Meta-Analyses (PRISMA) guidelines using the electronic database MEDLINE. The search was set to identify publications from 2003 to 2023 that focused on Chiari malformation and syringomyelia. The search included books, documents, clinical trials, meta-analyses, randomized control trials, and systemic reviews. We searched the database using the specific terms "Chiari-Malformation”, “Chiari type 1 malformation” and “Chiari malformation" to relate to the theme of Chiari malformation. We combined these search terms with the word “Syringomyelia” using the Boolean operator "AND". We also performed a more detailed search using the specific words “Pathogenesis”, “Pathophysiology”, “Diagnosis”, “Anatomical”, “Treatment” and “Surgical” again with the Boolean operator “AND” with the overall search term “Chiari malformation and syringomyelia.” Publications were included if they were written in English and the article's focus related to an aspect of Chiari malformation and syringomyelia. Articles were excluded if the inclusion criteria were not met. A total of 108 articles were excluded, including duplicate publications, unavailable articles, and those that did not follow the inclusion criteria (Figure 2).

Two authors (PS and NA) independently screened all retrieved items by title and abstract, then full text as necessary using the predetermined selection criteria. Disagreements were resolved through discussion (Greenhalgh and Peacock 2005). Data on article characteristics, design, classification, anatomical pathogenesis, treatment options, and prognosis were extracted by a single author (PS) from studies in a spreadsheet. Statistical analysis was performed using IBM SPSS Statistics for Windows, Version 28 (Released 2021; IBM Corp., Armonk, New York, United States). The primary outcome evaluated in this review was an anatomical understanding of Chiari malformation and Syringomyelia. Analysis was achieved through an iterative narrative synthesis process (Aveyard et al. 2016) [6,7].

## Discussion

Hans Chiari’s initial description of “hydrocephalus of the cerebrum” is one of a variety of theories discussed as a suggested pathogenic basis for the development of Chiari malformation. Both Gardner [8] and McLone et al. [9] proposed the importance of CSF drainage in posterior fossa development, although McLone et al. particularly focused their theory on children born with Chiari-type 2 malformation and myelomeningocele. Weed LH showed physiologically that fluid filtration through the neural tube and primitive fourth ventricle produces enough fluid and filtration pressure to cause opening of the subarachnoid space [10]. Gardner, using this, proposed that failure of adequate perforation of the membranous roof of the primitive fourth ventricle results in a pathological hydrocephalus that results in the displacement of the tentorium and a mismatch in CSF pulsation opening the central spinal canal. This causes a shallow posterior fossa development with hindbrain herniation [8]. However, McLone et al. proposed dysfunction earlier on in embryology specifically in primary neurulation is the issue. They said dysfunctional primary neurulation causes incomplete neural tube closure (resulting in a meningocele), predisposing CSF to drain straight into the intrauterine environment through the spinal defect subsiding the fourth ventricle. This results in ventricular collapse due to reduced CSF pulse pressure and a resulting hypoplastic posterior fossa and cerebellar tonsillar herniation [9]. Although difficult to compare given that the theory by McLone et al. was focused only on Chiari type 2 children with myelomeningocele, it is interesting to see how both increased/mismatched CSF pulse pressure and flow, in contrast to reduced CSF pressure and flow, have both been described for dysfunctional posterior fossa development.

In 1896, Hans Chiari subsequently described another pathogenic mechanism for Chiari malformation. He suggested that insufficiency in posterior fossa bone growth causes overcrowding and thus increases intracranial pressure pushing the cerebellar tonsils through the foramen magnum [1]. Numerous studies have also been performed highlighting the association of a small posterior fossa with Chiari type 1 malformation. An example of a study by Stovner et al. measured skull dimensions of patients on lateral skull radiographs in 33 patients with Chiari malformation and 40 control subjects [11]. The posterior cranial fossa was found to be significantly smaller and shallower in patients with Chiari malformation and in fact, there was a positive correlation between the size of the posterior cranial fossa and the level of cerebellar tonsillar herniation concluding that a poor size development of the posterior cranial fossa resulted in hindbrain herniation [11]. The underlying basis of insufficient posterior fossa growth is suggested by Marina-Padilla through primary paraxial mesoderm insufficiency results in occipital somite development dysfunction thus preventing neural fold closure. They supported this through experimental studies in which they gave vitamin A to experimental models to induce mesodermal insufficiency during development. This subsequently resulted in the induction of Chiari malformation in the experimental models suggesting a key role in primary paraxial mesodermal insufficiency in improper posterior fossa development [12].

It is interesting to note in this case illustration that the patient presented significantly with symptoms from syrinx compression but was relatively asymptomatic from a Chiari malformation point of view. This is common with Chiari type 1 malformation as even though it is symptomatic in 1 in 1000 people, 1 in 100 actually meets the radiological criteria for diagnosis. A study by Sadler et al. showed that Chiari type 1 patients who typically present early are associated with genetic and multiple congenital abnormalities. Therefore, although Chiari malformation is common, patients rarely present unless associated with significant co-morbidities, severe presentations, or complications of the hindbrain abnormality [13].

Significant risk factors suggested for the development of a syrinx in Chiari malformation patients include tonsillar herniation of more than 12mm, sagittal diameter of the foramen magnum of more than 0.6mm, and a clival angle of less than 0.8mm [2]. These variables were put together by a statistician following a study in Birmingham looking at the cranial metric measurements of the posterior cranial fossa. These demonstrated findings suggest that a small posterior fossa may play a role in syrinx formation in patients with Chiari malformation due to cerebellar descent and obstruction of CSF flow at the level of the foramen magnum. In saying that, multiple other theories exist that also discuss other pathogenic mechanisms relating to syrinx development in Chiari malformation.

In 1958, Gardner proposed the “Water Hammer theory.” He used the basis of his theory on the failure of adequate perforation of the membranous roof of the fourth ventricle and obstruction of CSF outflow from the fourth ventricle. During systole, arterial pulsation of the ventricular fluid occurs causing increased fluid out of the ventricles. However, due to the pathological blockage of the outlets of the fourth ventricle, CSF is pushed directly into the central canal of the spinal cord. The increase in CSF volume within the patent central canal results in dilation of the ventral canal and syrinx formation [8]. Over time, general acceptance of this theory has been limited as the pathological blockage of CSF outflow through the fourth ventricle as proposed by Gardner should also result in hydrocephalus. However, multiple cases exist, including that illustrated in my patient, of those who develop syringomyelia in the absence of hydrocephalus. Furthermore, based on this theory suboccipital craniectomy with plugging of the obex became common and was initially thought to be effective [14]. However, between 1998 and 2001, a study was performed by Alden et al. which examined 21 patients surgically treated with Chiari malformation, of which 10 patients had syringomyelia [15]. The surgical approaches performed included a limited suboccipital craniectomy with C1 laminectomy +/- scoring of the dura, durotomy, duroplasty, intradural exploration, or resection of the cerebellar tonsils with duroplasty. Out of the 10 patients with syringomyelia, these surgical procedures resulted in the resolution in eight patients, with one patient remaining the same and one where the size of the syrinx decreased [15].

Oldfield et al. also describe an increase in spinal surface pressure contributing to syrinx formation and progression, but unlike Gardner who describes this increase in pressure due to increased CSF volume within the central cord of the spinal cord, Oldfield proposes this increase in pressure as secondary to the rapid tonsillar movement against an entrapped spinal space. Physiologically, he describes that CSF usually moves from the intracranial space into the spinal subarachnoid space during systole as the brain fills with blood during systole and expands. However, hindbrain herniation in Chiari malformation prevents this movement, and therefore, the rapid downward movement of the cerebellar tonsils occurs to accommodate for this absent CSF movement. The piston-like movement of the cerebellar tonsils will create a CSF pressure wave that contracts the cord forcing fluid into it [1,16]. This is supported by a study performed by Heiss et al. (Oldfield) using 20 adult patients with Chiari 1 malformation and symptomatic syringomyelia compared with 18 healthy volunteers where testing was done pre-surgically, during surgery, and post-surgically [17]. Pre-surgically he found that CSF velocity was increased at the foramen magnum, but the flow of CSF was reduced due to the decreased AP diameter of the CSF space at the foramen magnum. He also found that intracranial pressure transmission to the spinal subarachnoid space was obstructed and during surgery, the cerebellar tonsils would abruptly descend during systole which was matched with the movement of the upper pole of the syrinx. Overall, he found that following surgery as the diameter of the foramen magnum increased the maximum flow rate of CSF during systole increased. The transmission of intracranial pressure to the spinal subarachnoid space was normal resulting in the syrinx diameter decrease in all patients [17]. However, it is difficult to explain how such piston-like movement resulting in contraction of the cord and increased pressure can result in such significant fluid movement to cause syrinx formation if the cord was so contracted [18].

The origin of syringomyelia fluid in Chiari malformation is also widely discussed where Koyanagi et al. and Greitz propose that syringomyelia formation occurs through extracellular fluid accumulation through various pathological mechanisms as opposed to CSF accumulation. 

Koyanagi et al. proposed that extracellular fluid accumulation contributes to syringomyelia formation through disturbed absorption from intramedullary venous channels. They highlight the importance of compliance in precipitating dysfunction in the role of intramedullary venous channels. Compliance is defined by Williams as the ability of the body to enable a volume increase in the intrathecal CNS space without causing a pressure increase. Physiologically the venous system is important for managing compliance within the CNS as the veins are highly compressible and capable of large volume fluctuations to maintain pressure. In Chiari malformation, obstruction of the CSF flow due to a small posterior fossa and hindbrain herniation causes a state of reduced spinal compliance due to a narrowed cervical spinal space from tonsillar herniation. The inability of the spinal space to accommodate abrupt changes in volume is reduced [19]. Due to the location of the posterior spinal vein situation in the posterior pial surface of the subarachnoid space, it is directly influenced by reduced spinal subarachnoid space compliance, and large fluctuations in pressure occur to compensate for this. This disrupts autoregulation and absorption of extracellular fluid through the intramedullary venous channels [20]. However, Greitz uses the principle of the venturi effect as opposed to reduced compliance resulting in extracellular fluid accumulation. The venturi effect highlights that a regional increase in fluid velocity within a narrow channel will result in a decrease in fluid pressure. He explains that in Chiari malformation there is a localized increase in CSF pulse velocity due to obstruction of CSF flow at the level of foramen magnum, with a narrowing of the cervical spinal canal due to hindbrain herniation. This regional increase in CSF velocity will result in a low fluid pressure as seen through the Venturi effect. The low fluid pressure within the spinal cord will cause a suction effect on the spinal cord that distends. Extracellular fluid located within the cavities and channels within the spinal cord accumulates due to this suction effect within the cord distention. The dilated caudal section of the syrinx, associated with a decreased CSF pressure in the adjacent subarachnoid shows a transient pressure gradient allowing the syrinx to expand [21]. Overall, the effect of flow obstruction and pressure decrease causes an extracellular fluid build-up within the cord predisposing to syrinx formation.

The role of extracellular fluid accumulation resulting in syrinx formation is still debated whether that is through reduced spinal compliance from venous dysfunction as stated by Koyanagi et al. or a low fluid pressure within the spinal cord causing a suction effect as described by Greitz. A key to establishing whether extracellular fluid accumulation has a major role in syrinx development is by looking at how patients with syringomyelia and Chiari malformation are typically treated and their treatment outcomes. As we have seen previously suboccipital craniectomy, C1 laminectomy, and duraplasty have shown great clinical outcomes in treated patients, however, it is important to also assess its clinical outcomes and benefits with other procedures also used. Iwasaki et al. performed a study on 80 patients with syringomyelia with Chiari malformation who were surgically treated. They analyzed two major procedures including foramen magnum decompression involving craniectomy of the posterior fossa, C1 laminectomy, and resection of the epidural band with little intradural exploration as well as the placement of a syringe-subarachnoid shunt. A comparison of these two procedures showed significant short-term clinical neurological improvement in the syringe-subarachnoid shunt procedure compared to the foramen magnum decompression group. The placement of the shunt into the syrinx and into the spinal subarachnoid space allowed a route for CSF movement across the cord during cardiac systole [22]. Although associated with good short-term clinical outcomes shunt placement would not be beneficial in the long term due to its risk of occlusion, infection, and malposition. Overall, although the clinical benefits of shunt placement generally outweigh its risks, its insertion should be adequately assessed with the patient's clinical symptoms, performance status, and severity of Chiari malformation. 

However, we can use the clinical benefit achieved with shunt placement to highlight accuracy in the proposed theories of Koyanagi et al. and Greitz [20,21]. Using Koyanagi et al. proposed theory of reduced compliance we can see the placement of a shunt allows CSF compliance to be increased as the shunt allows a passage for CSF flow. This causes the syrinx pressure to decrease and the ability of the spinal space to accommodate increased CSF volume relative to the upper cervical narrowing. This allows physiological venous compensation and extracellular fluid to be absorbed normally through the intramedullary venous channels. The venturi principle as proposed by Greitz is slightly more difficult to support through the placement of a syringe-subarachnoid shunt, as it does not remove the cervical spinal narrowing from hindbrain herniation that causes the low spinal pressure and accumulation of extracellular fluid from the cavities and channels. It also creates a further passage for CSF flow, further decreasing the fluid pressure within the spinal cord.

A craniospinal pressure dissociation is another proposed theory by Williams. He noted that the pressure between the spine and the brain at rest is equal however on coughing or performing a Valsalva there would be a raised ventricular pressure resulting in a positive deflective tract causing rapid CSF movement. He found that this pressure was also the same in patients with hindbrain herniation like that seen in Chiari malformation during systole when performing a Valsalva. Like Greitz he also described a ball valve effect where hindbrain herniation would result in blockage at the level of the foramen magnum. As fluid is unable to enter through the foramen magnum it is forced into the central canal from the increased pressure generated during the peak of craniospinal dissociation causing a suck of fluid from the ventricle into the syrinx during systole. Extension of the syrinx cavity is seen through pressure differences and fluid shifts through fluid movement during raised intracranial pressure which he described as “slosh” [23]. Dynamic MRI imaging performed by Honey et al. showed evidence of extension of syrinx extension through “slosh” [18]. They found that CSF pulse pressure was greatest at systole when performing a Valsalva and this would result in asymmetric contraction of the syrinx rostrally causing rapid movement of the syrinx fluid caudally causing distention of the inferior portion of the syrinx. They found that subsequently during diastole as pressure reversed fluid would slowly move to its original position with normalization of the lower portion of the syrinx with some fluid also moving rostrally to extend the fluid upward. These images highlight the pressure differences described by Williams and their influence on syrinx progression [18].

Chang et al. also highlight the role of a small posterior fossa in predisposing to syrinx development, as they suggest that overcrowding of the posterior fossa in Chiari malformation results in cisterna manga dysfunction [14]. CSF usually drains into the cisterna magna from the fourth ventricle however as this is reduced, the CSF pressure through the central canal is increased due to the increasing fluid draining through this route. The pathological basis of this theory can be thought similar to that proposed by Gardner who states that fluid is pushed into the central canal due to the pathological blockage of the fourth ventricle. The resultant dysfunction of the cisterna magna and increased fluid into the central canal results in pressure being markedly increased leading to the leakage of the CSF into the parenchyma and thus formation of the syrinx. Compared to Gardner, Chang uses the basis of a small posterior fossa and overcrowding as the basis of their theory which, as seen above, is supported by several studies. They describe key objections in their theory, such as assuming the patency of the central canal and other communication channels between the fourth ventricle and the syrinx when physiologically the central canal will have some degree of stenosis as people age and communications are often non-existent on MRI. In light of this, Chang goes on to state that although MRI imaging has come a long way over the years, there is still a relative limitation in the resolution of imaging, and it may not always detect the complete patency of the central canal or communication channels. They also note that not all patients with Chiari malformation and syringomyelia will be old and therefore will often present prior to the physiological process of central canal stenosis in old age [14].

Furthermore, the creation of an artificially enlarged cisterna magna is performed in patients having a suboccipital craniectomy, C1 laminectomy, and duraplasty for the treatment of syringomyelia in Chiari patients. Therefore, given the clinical effectiveness of this procedure in removing the mechanical obstruction of CSF flow at the foramen magnum, it is also likely that the achievement of an artificially enlarged cisterna magna also plays a role in the clinical effectiveness of this procedure suggesting that dysfunction of the cisterna magna may play a contributing factor to syringomyelia. To establish such a link, it is necessary to perform more detailed studies in the future highlighting the relationship between syringomyelia severity and association with the size of the posterior fossa. If results were to show those with more extensive syringomyelia were associated with smaller posterior fossa, this could suggest that the level of cisterna magna dysfunction is much more prominent in these patients and therefore plays a key role in syringomyelia development in these patients.

## Conclusions

In conclusion, there is growing evidence for various pathological mechanisms describing syringomyelia and Chiari malformation. The evidence basis for all theories is continually developing; however, we know from numerous studies that a small posterior fossa is seen in patients with Chiari malformation. A small posterior fossa and hindbrain herniation most definitely play a role in mechanical obstruction of CSF flow at the level of the foramen magnum; however, whether this contributes solely to syringomyelia is still discussed. Furthermore, with this theory, it is difficult to note the presence of syringomyelia without the presence of hydrocephalus as noted in my case illustration therefore suggesting further pathological mechanisms also contribute. It is likely a small posterior fossa contributing to other anatomical abnormalities such as cisterna magna dysfunction, cerebellar tonsils entrapment within the spinal space, and craniospinal pressure dissociation plays a major role in syringomyelia development. This suggests that syrinx development is multifactorial and is a result of the anatomical abnormalities seen in Chiari malformation.

## References

[1] Buell TJ, Heiss JD, Oldfield EH (2015). Pathogenesis and cerebrospinal fluid hydrodynamics of the Chiari I malformation. Neurosurg Clin N Am.

[2] (2002). Greenfield’s Neuropathology. Greenfield’s Neuropathology, 7th Ed, volume 1. (D.L Graham, Ed) London & New York : Arnold.

[3] (2009). Brain’s Diseases of the Nervous System Twelfth Edition.

[4] Bordes S, Jenkins S, Tubbs RS (2019). Defining, diagnosing, clarifying, and classifying the Chiari I malformations. Childs Nerv Syst.

[5] Hiremath SB, Fitsiori A, Boto J (2020). The perplexity surrounding Chiari malformations - are we any wiser now?. Am J Neuroradiol.

[6] Page MJ, McKenzie JE, Bossuyt PM (2021). The PRISMA 2020 statement: an updated guideline for reporting systematic reviews. BMJ.

[7] Aveyard H, Payne S, Preston N (2016). A Post-graduate’s Guide to Doing a Literature Review in Health and Social Care. https://books.google.co.in/books?id=AMkvEAAAQBAJ&printsec=frontcover&source=gbs_ge_summary_r&cad=0#v=onepage&q&f=false.

[8] Gardner WJ (1965). Hydrodynamic mechanism of syringomyelia: its relationship to myelocele. J Neurol Neurosurg Psychiatry.

[9] McLone DG, Knepper PA (1989). The cause of Chiari II malformation: a unified theory. Pediatr Neurosci.

[10] Weed LH (1917). The development of the cerebro-spinal spaces in pig and in man. Contr Embryol Carneg.

[11] Stovner LJ, Bergan U, Nilsen G, Sjaastad O (1993). Posterior cranial fossa dimensions in the Chiari I malformation: relation to pathogenesis and clinical presentation. Neuroradiology.

[12] Marin-Padilla M, Marin-Padilla TM (1981). Morphogenesis of experimentally induced Arnold-Chiari malformation. J Neurol Sci.

[13] Sadler B, Kuensting T, Strahle J (2020). Prevalence and impact of underlying diagnosis and comorbidities on Chiari 1 malformation. Pediatr Neurol.

[14] Chang HS, Nakagawa H (2003). Hypothesis on the pathophysiology of syringomyelia based on simulation of cerebrospinal fluid dynamics. J Neurol Neurosurg Psychiatry.

[15] Alden TD, Ojemann JG, Park TS (2001). Surgical treatment of Chiari I malformation: indications and approaches. Neurosurg Focus.

[16] Oldfield EH, Muraszko K, Shawker TH, Patronas NJ (1994). Pathophysiology of syringomyelia associated with Chiari I malformation of the cerebellar tonsils. Implications for diagnosis and treatment. J Neurosurg.

[17] Heiss JD, Patronas N, DeVroom HL (1999). Elucidating the pathophysiology of syringomyelia. J Neurosurg.

[18] Honey CM, Martin KW, Heran MK (2017). Syringomyelia fluid dynamics and cord motion revealed by serendipitous null point artifacts during cine MRI. AJNR Am J Neuroradiol.

[19] Williams H (2008). A unifying hypothesis for hydrocephalus, Chiari malformation, syringomyelia, anencephaly and spina bifida. Cerebrospinal Fluid Res.

[20] Koyanagi I, Houkin K (2010). Pathogenesis of syringomyelia associated with Chiari type 1 malformation: review of evidences and proposal of a new hypothesis. Neurosurg Rev.

[21] Greitz D (2006). Unraveling the riddle of syringomyelia. Neurosurg Rev.

[22] Iwasaki Y, Hida K, Koyanagi I, Kuroda S, Abe H (1995). Surgical treatment for syringomyelia associated with Chiari malformation (Article in Japanese). Rinsho Shinkeigaku.

[23] Williams B (1980). On the pathogenesis of syringomyelia: a review. J R Soc Med.

